# Osteoporosis in Patients With Marfan Syndrome: A Narrative Review of Bone Health and Management

**DOI:** 10.7759/cureus.99172

**Published:** 2025-12-13

**Authors:** Abdul Waheed Bahir, Munir Ahmad Bahir, Qudratullah Bahir, Gu Shao, Xiong Ying

**Affiliations:** 1 Department of Orthopedic Surgery, Yan’an Hospital Affiliated to Kunming Medical University, Kunming, CHN; 2 Department of Clinical Medicine, Malalay University, Kandahar, AFG

**Keywords:** bone density, connective tissue disorder, fbn1 mutation, fracture risk, marfan syndrome, osteoporosis

## Abstract

Marfan syndrome is a hereditary connective tissue disorder that is caused by pathogenic variants in the FBN1 gene and is traditionally known to have cardiovascular and ocular presentations. There has been growing data showing that bone quality impairment and decreased bone mineral density are significant but undervalued factors of the illness. Osteoporosis in patients with Marfan syndrome can develop earlier, follow different pathways, and lead to an increased risk of fragility fractures in comparison with those in the general population. The present narrative review is a critical evaluation of the existing knowledge of bone health in Marfan syndrome and includes the underlying molecular pathways of skeletal fragility, the impact of skeletal abnormalities and biomechanical changes, and how growth-factor malregulation can drive bone remodeling. Diagnostic analysis has been difficult given the overlapping musculoskeletal abnormalities, and requires a holistic evaluation including clinical examination, and imaging on cases like the dual-energy X-ray absorptiometry, and genetic analysis where necessary. Traditional treatments of osteoporosis can increase bone density, but not completely correct the connective tissue defects or disease-specific determinants of bone weakness. On the other hand, pharmacologic agents that are regularly used in the treatment of cardiovascular protection in Marfan syndrome have little to contribute to bone health, which highlights the urgency of skeletal-specific therapeutic interventions. The risk of fractures and the importance of reducing it, as well as maintenance of long-term musculoskeletal function, require early screening, individualized management, and multidisciplinary care. Anatomical and biomechanical factors that cause osteoporosis in this at-risk group are unique, and further studies are needed to design therapeutic approaches that would respond to these factors.

## Introduction and background

Osteoporosis is a bone disorder characterized by the loss of bone mass and bone microarchitecture deterioration, leading to increased bone fragility and a higher susceptibility to fractures [1]. Although commonly coupled with the aging process, osteoporosis is also seen in people with genetic connective tissue disorders, such as Marfan syndrome [2]. Marfan syndrome is an autosomal dominant syndrome that involves variants in the FBN1 gene pathogenic to biological activities that cause the synthesis of fibrillin-1, which is a fundamental structural protein involved in connective tissue integrity in all parts of the body [3]. These molecular defects play a role not only in the established cardiovascular and ocular morbidity of the syndrome but also in the skeletal development and bone disease [4]. Common approaches to the management of osteoporosis include lifestyle changes, nutritional supplementation, and pharmacological treatment [5]. The effectiveness of antiresorptive agents continues to lie in the foundation of osteoporosis management, having proven effective in decreasing the bone turnover and risk of fractures, although therapeutic responsiveness across skeletal sites can be difficult, and bone mineral density (BMD) increases do not always lead to an increase in bone strength [6]. An example is that bowing alendronate could enhance the quality of the bone matrix with osteopenia and lordosis, but it has no effect on the evolution of aortic aneurysms, one of the cardiovascular outcomes that are the most detrimental manifestations of Marfan syndrome [7]. On the other hand, losartan, an agent that suppresses TGF 2 mounting and strengthens the aortic wall in severe cases of Marfan syndrome, does not prevent bone insurgency, thus highlighting the need for skeletal-specific treatment strategies in this group [8]. Osteoporosis is often referred to as a silent thief because it has an insidious progression until the occurrence of a fracture [9]. Even though it can be frequently seen among post-menopausal women and older men [10], this syndrome poses a specific clinical problem in patients with Marfan syndrome. Typical skeletal phenotypes such as long extremities, spinal defects, and defects in the chest wall, combined with impaired bone quality, could increase the rate of fracture and may contribute to skeletal and cardiovascular adverse events [4]. This study underscores the need to note osteoporosis as a comorbidity that has not been given serious consideration in Marfan syndrome and the need to promote early assessments and tailored interventions. To support this narrative review, a thorough literature search was conducted using PubMed, Google Scholar, and Cochrane Library databases. Among the keywords used were osteoporosis, bone density, fracture risk, Marfan syndrome, FBN1 mutation, and connective tissue disorder, preparing and combining them in varying formulations. Publications in the English language that covered bone health in relation to Marfan syndrome were prioritized. This methodological approach helped in the synthesis of the heterogeneous findings, hence giving an overall picture of osteoporosis in patients with Marfan syndrome.

## Review

Pathophysiology of Marfan syndrome

Marfan syndrome is a hereditary connective tissue disorder mostly due to mutations in the FBN1 gene, encoding fibrillin-1, a major component of the extracellular matrix [11]. The mutations destabilize the structure and functioning of fibrillin-1, leading to impaired microfibril formation and disordered connective tissue organization. Fibrillin-1 plays a primary role in supporting the structure and elasticity of the skeletal, cardiovascular, and ocular systems [12]. Such widespread distribution of fibrillin-1 is the result of the systemic nature of Marfan syndrome. In the musculoskeletal system, there are abnormalities of connective tissue that tend to bring about long limbs, scoliosis of the spine, and pectus excavatum of the chest wall [13]. All of these skeletal distortions point to a structural weakness in people that puts them at risk of having slack joints, postural abnormalities, and a high risk of fractures. FBN1 mutations are associated with the cardiovascular system, aortic wall weakening resulting in dilation of the aortic root, development of aneurysms, and potentially fatal aortic dissection. The ocular symptoms, such as dislocation of the lens and myopia, are additional examples of the widespread effect of fibrillin-1 deficiency and the support of the systemic activity of connective tissues in Marfan syndrome [14].

Skeletal manifestations

The skeletal abnormalities are key characteristics of the Marfan syndrome and the central clinical phenotype [15]. Patients commonly have disproportionate limb lengthening and long arms, legs, fingers, and toes, which can be explained by laxity of the connective tissue and other changes in the biomechanical forces applied to the developing bone mass [13]. Popular spinal defects include scoliosis, kyphosis, and severe lumbar lordosis, which can lead to pain, post-spinal instability, and, in extreme cases, erode pulmonary functionality [16]. Anatomy Discontinuities such as pectus excavatum and pectus carinatum are manifestations of structural weakness of the costal cartilage and sternum; they can cause cosmetic issues and, in a few cases, result in cardiopulmonary restrictions [17]. Other common aspects include joint hypermobility, which is a result of ligamentous laxity; excessive range of motion increases the risk of instability, recurrent subluxations or dislocation, and long-term musculoskeletal pain, thus hindering mobility and general quality of life [18]. Importantly, osteoporosis and fragility fractures are associated with a high risk in Marfan syndrome, probably because of inherent bone defects, low physical activity, and as a complication in some instances, because of chronic exposure to glucocorticoids [19]. These combinations are synergistic and decrease the strength of the bones and increase the risk of fractures.

Osteoporosis in Marfan syndrome

Osteoporosis is a systemic bone skeletal disease characterized by bone mass and microarchitectural bone destruction that leads to increased bone fragility and fracture. In the framework of Marfan syndrome, osteoporosis has become a relevant complication woven into connective tissue defects and the genetic factors [2]. People affected by Marfan syndrome usually have low BMD compared to average people, and hence this predisposes them to fractures after low-energy mechanical injuries [20]. Epidemiological data further support this increased skeletal vulnerability. Studies in children with Marfan syndrome demonstrate height-adjusted whole-body and lumbar spine BMD Z-scores reduced by approximately 0.8 SD compared with matched controls, indicating a mild but generalized deficit in bone mass [2]. In addition, a nationwide Danish cohort study involving 406 patients with Marfan syndrome reported that 21.9% had experienced at least one fracture, compared with 18.9% of matched controls, and that 10.3% met composite criteria for osteoporosis, compared with 3.3% in the reference population [19]. These findings highlight that reduced BMD and fracture risk are both clinically meaningful and disproportionately elevated in this population.

**Figure 1:**
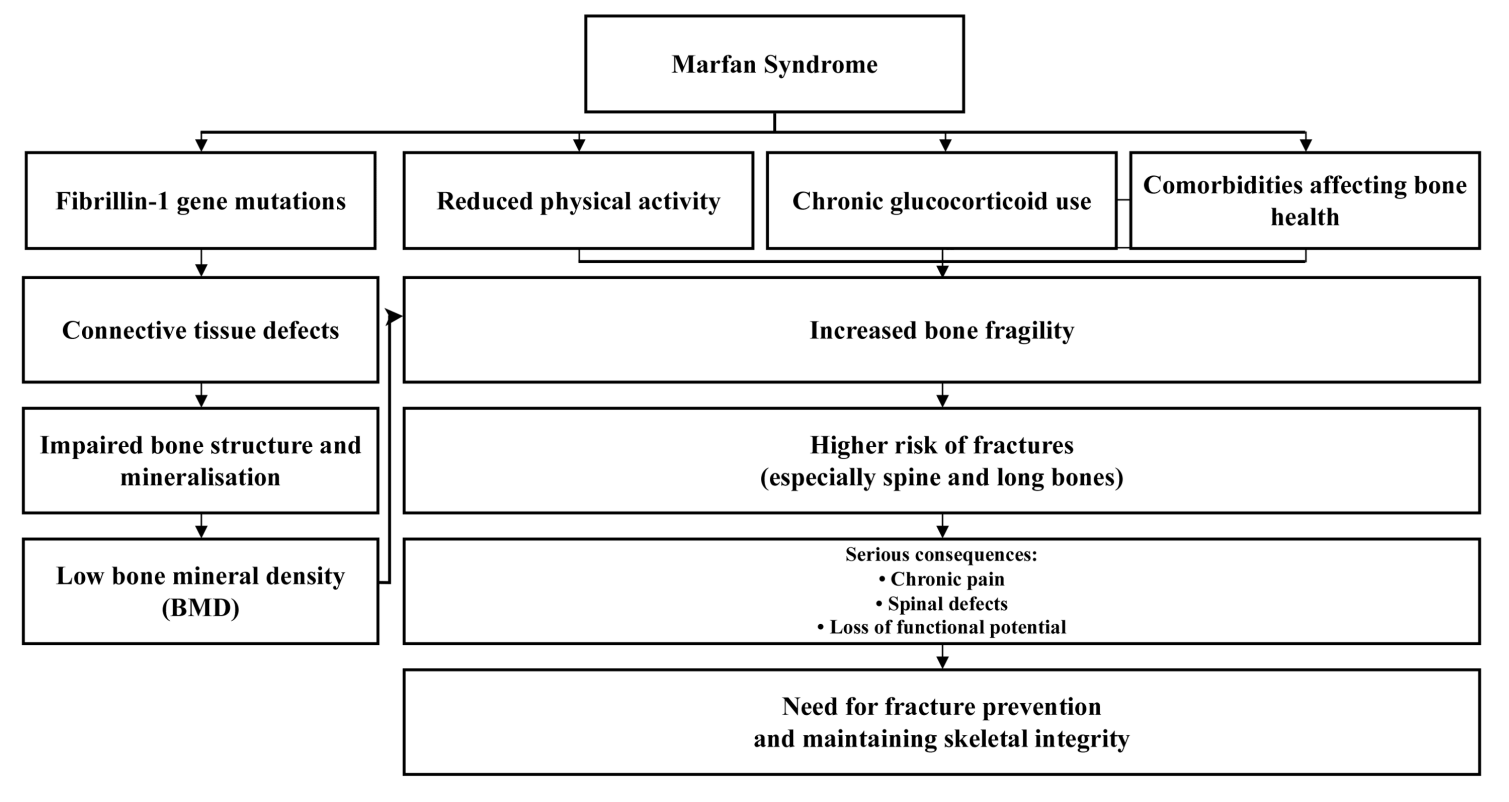
Osteoporosis Pathway in Marfan Syndrome

The resultant structural compromise, particularly in the vertebral column and long bones, is a consequence of affected bone quantity, in addition to the quality. Deformed bone structure and mineralization deficiency are mutations of the fibrillin-1 gene, which weaken the bones and increase their susceptibility to fractures [21]. Additional contributory factors include a reduction in physical activity, persistent intake of glucocorticoids, and supportive comorbidities, which adversely affect skeletal health [22]. A fracture of the spine and long bones in patients with Marfan syndrome can cause critical consequences, such as chronic pain, spinal defect, and loss of functional potential. Therefore, maintenance of skeletal integrity and fracture prevention are among the main goals in the management of osteoporosis in this group (Figure 1) [23].

Genetic links and impact on bone health

**Figure 2:**
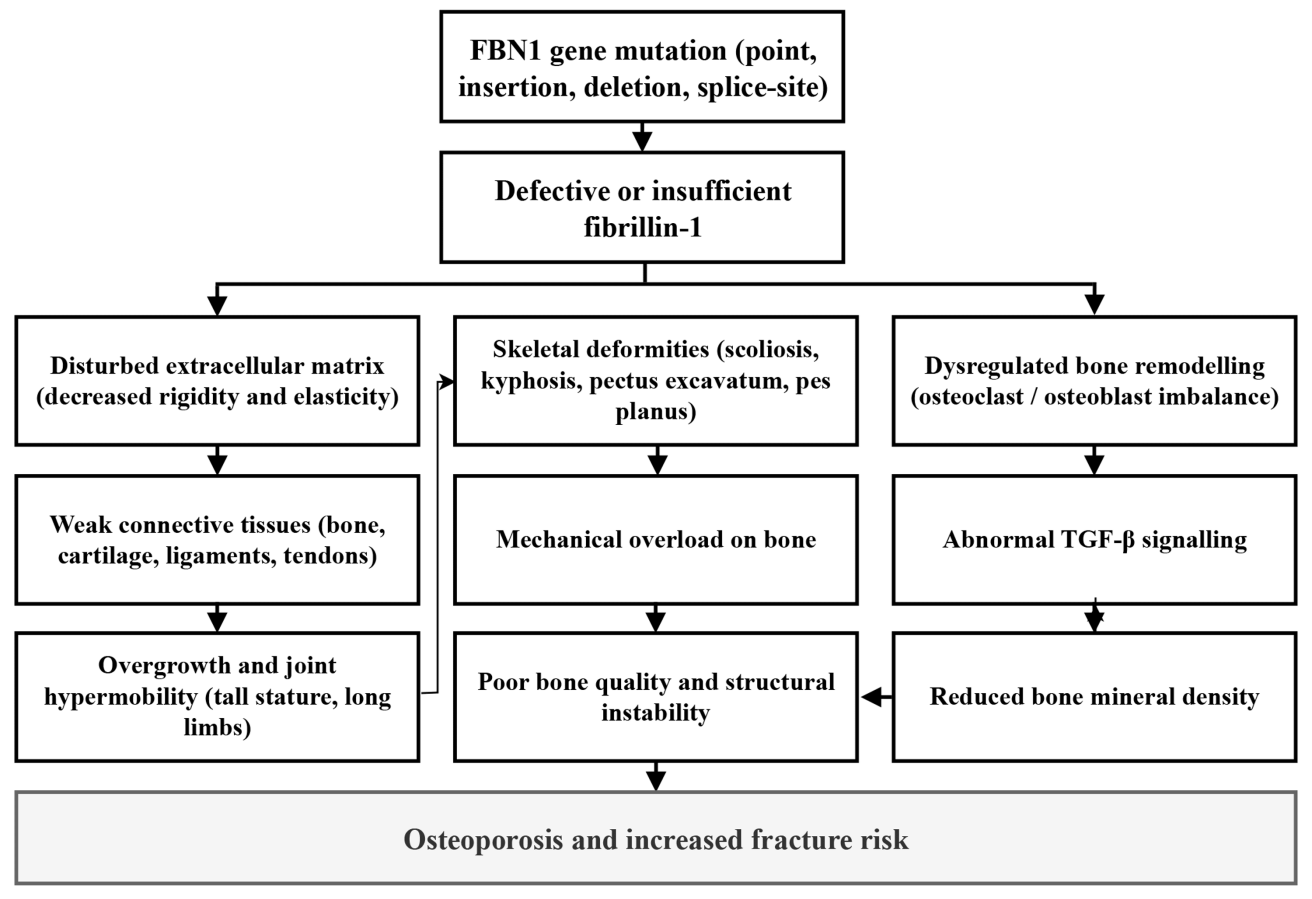
How Marfan Syndrome Leads to Weak Bones and Fractures

The FBN1 gene is the main causative agent of Marfan syndrome, and its mutations cause quantitative or qualitative deficiencies in fibrillin-1 [24]. These pathologic changes include a point mutation, insertions, deletions, and splice-site mutations, each of which could disrupt the biosynthesis or functional integrity of fibrillin-1. Due to the central structural position of fibrillin-1 in the extracellular matrix of connective tissue, its distortion leads to under-rigidity and decreased elasticity of tissues across the organism. Fibrillin-1 plays a critical role in maintaining the mechanical stability and integrity of connective tissues that maintain skeletal structures, such as bone, cartilage, ligaments, and tendons [12]. Connective tissues are also very sensitive to overstretching and deformation when the fibrillin-1 functions are impaired. This phenomenon is clinically manifested in the form of an increase in stature, overgrowth of limbs, joint hypermobility, and a distinctive body habitus of the Marfan syndrome. Dysregulated bone remodeling is also a threat to bone health. Normative bone remodeling also requires a balanced relationship between osteoclastic-mediated resorption and osteoblast-mediated formation [25]. In Marfan syndrome, this balance could be disrupted by perturbations in fibrillin-1 and other signaling cascades, such as TGF-β, and ends with disrupted bone formation, diminished BMD, and increased likelihood of fractures [26]. Fibrillin-1 also plays a role in bone development and mineralization under normal conditions. Its activity is disrupted, affecting coordinated ontogenetic events in development, resulting in skeletal deformities such as scoliosis, kyphosis, pectus excavatum, and pes planus [27]. Not only do these skeletal deformities modify external morphology, but they also subject the bone to aberrant mechanical loads even greater to reduce bone quality and structural stability [28]. The combination of genetic aberrations, disturbed remodeling processes, and mechanical forces creates a biological and biomechanical environment that predisposes patients to osteoporosis and fracture in Marfan syndrome (Figure 2).

Diagnostic considerations in Marfan-related osteoporosis

In patients with Marfan syndrome, osteoporosis needs to be carefully diagnosed after careful exclusion of symptoms due to deformities and actual bone fragility. The connective tissue defects caused by fibrillin-1 contribute to skeletal malformation (i.e., lengthening of the limbs, kyphosis, and deformity of the chest wall) [29], and osteoporosis results in fractures of the bone, bone pains, and defects. An in-depth examination should include all the clinical results, imaging, and assessment of the risk factors. Musculoskeletal instability and frequent joint injury are also associated with joint hypermobility, which is typical of Marfan syndrome and is caused by laxity of the connective tissues [30]. Medical instruments such as the Beighton score may be used to measure hypermobility and differentiate between pathological laxity of the joint and benign ones [31]. Osteoporosis does not cause joint hypermobility, but the subsequent instability and lack of physical activity have the indirect effect of deteriorating bone health. FBN1 mutations are genetic tests that can play an essential role in the diagnosis of Marfan syndrome, especially in patients with either overlapping or atypical phenotypes [28]. Identification of a pathogenic variant assists in differentiating Marfan syndrome from any other hereditary connective tissue disorder and helps in the early surveillance and preventive control. Imaging research is important for determining bone health. The most common form of measuring BMD in the diagnosis of osteoporosis or osteopenia is dual-energy X-ray absorptiometry (DXA) [32]. Further radiographic visuals could capture vertebral fractures or defects, which otherwise may be treated in a clinical manner. Skeletal, ocular, cardiovascular, and genetic characteristics are combined in clinical diagnostic models such as the Ghent criteria, which identify a diagnosis of Marfan syndrome and direct its management [33]. A careful physical examination is vital, and the proportions of the limbs, spinal position, chest wall structure, joint stability, and any evidence of cardiovascular compromise, including murmurs or aortic root dilation, are considered [34]. Simultaneously, laboratory tests, such as serum calcium, vitamin D, and bone-specific alkaline phosphatase, are used to evaluate bone metabolism and determine the correctable factors of low BMD. Among these, serum tartrate-resistant acid phosphatase 5b (TRACP-5b) is a highly specific marker of osteoclastic bone resorption, correlates strongly with osteoclast number, and is not influenced by renal function. Including TRACP-5b can therefore enhance the evaluation of bone turnover in Marfan-related osteoporosis and assist in monitoring therapeutic response [35]. Bone turnover measurements and, in some cases, sophisticated genetic measurements could help in stratifying the risk and monitoring therapy response [36].

Clinical implications and future directions

The coexistence of Marfan syndrome and osteoporosis is a matter of significant diagnostic and treatment concerns. Early indicators of bone frailty may be obfuscated by overlapping skeletal abnormalities, and this is where proactive screening and routine BMD evaluation are essential in this potential high-risk group [19][37]. This necessitates an interdisciplinary solution, and the interdisciplinary team to use is cardiology, orthopedics, endocrinology, genetics, and rehabilitation medicine [38,39]. One-to-one management ought to consider age, sex, severity of diseases, fracture history, cardiovascular status, as well as the tolerability of treatment. The main aspects of long-term care include lifestyle changes, an exercise regimen tailored to the cardiovascular impairments, and a cautious pharmacologic approach. Prospective treatments that are aimed at bone remodeling and signaling pathways, for example, anti-sclerostin treatment or other new bone-active drugs, can bring future benefits to patients with Marfan-related osteoporosis but have to be evaluated thoroughly in clinical trials [40,41]. However, because anti-sclerostin antibodies such as romosozumab have shown higher rates of serious cardiovascular adverse events in phase III trials compared with control treatments [42,43] and regulatory agencies have issued cardiovascular warnings based on these findings [44], and because no dedicated safety data exist for individuals with Marfan syndrome, their use in this population should be considered theoretical and approached with caution until adequately studied. Imaging and age-related developments in bone assessment methods could also improve early detection, risk classification, and monitoring of the response. Continuous cooperation between academic centers, clinicians, and patient groups will also be critical to enhance evidence-based interventions and implement research findings into more promising results for patients with Marfan syndrome and osteoporosis.

## Conclusions

Marfan syndrome refers to an inherited connective tissue disorder in which skeletal integrity is affected by the FBN1 defects and is prone to osteoporosis and fragility fractures. Skeletal deformities are associated with reduced bone mineral density, abnormal bone architecture, and mechanical stress, all of which increase the risk of fracture and impairment. Diagnosis relies on clinical criteria, imaging, genetic testing, and biochemical evaluation to accurately diagnose the disease. This narrative review has demonstrated the significance of osteoporosis as an important complication of Marfan syndrome and the necessity to treat it thoroughly in a personalized manner, which presupposes lifestyle changes, the use of pharmacologic treatment in case of need, and continuous multidisciplinary care. Existing interventions to maintain bone health, such as early detection and intervention, can mitigate the nightmares of bone fractures and enhance quality of life in this susceptible group.
